# Subject Specific Mastery Motivation in Moldovan Middle School Students

**DOI:** 10.3390/bs13020166

**Published:** 2023-02-14

**Authors:** Marcela Calchei, Tun Zaw Oo, Krisztián Józsa

**Affiliations:** 1 Institute of Education, University of Szeged, 6722 Szeged, Hungary; 2 MTA-MATE Early Childhood Research Group, 7400 Kaposvár, Hungary; 3 Institute of Education, Hungarian University of Agriculture and Life Sciences, 7400 Kaposvár, Hungary

**Keywords:** subject-specific mastery motivation, measurement invariance, latent mean differences, gender differences

## Abstract

Given the crucial role of mastery motivation in the cognitive development of children, the present study investigates subject-specific mastery motivation in the multilingual educational system of the Republic of Moldova. We applied cross-sectional data from fifth, seventh, and ninth graders studying either in the Romanian (n = 583) or Russian (n = 353) language using the Subject Specific Mastery Motivation Questionnaire (SSMMQ). To ensure the validity of the comparison of latent mean differences, the Romanian and Russian versions of SSMMQ were validated and measurement invariance of the constructs across language, grade, and gender was assessed. The full scalar invariance across grades and gender and the partial scalar invariance across language held. Thus, a comparison of latent mean differences across these three groups is plausible. The findings proved that there was no difference between the Romanian and Russian samples, but we found girls self-rated themselves significantly higher than boys in the Reading, Art, and Music mastery motivation scales. Results with respect to the comparison of latent mean differences between the grade levels demonstrated that the Reading mastery motivation of the Moldovan students stayed stable from fifth to ninth grades, whereas Art had a constant declining path.

## 1. Introduction

Mastery motivation is a person’s intrinsic urge that drives and maintains a behavior focused on mastering a challenging task, skill, or competence [1,2]. The exhibit of mastery motivation generates mastery pleasure in success, and a further drive to complete a challenging task. Empirical studies demonstrated that mastery motivation is a valid predictor of children’s social, cognitive, and psychomotor developments [3,4,5,6]. Mastery motivation mediates learning and school achievement in both formal and informal educational setups [1]. There is an extended body of research focusing on mastery motivation as an explanatory factor of various school achievement, engagement, psychological well-being, cognition, future professional choices, etc. [1,7,8,9,10,11,12,13,14,15,16]. Therefore, exploring the pattern of mastery motivational specificity in academic domains can explain the school achievement trajectories and further understand how students’ learning in different subjects can be supported by education stakeholders in school settings.

Although subject-specific mastery motivation has an important role in the academic trajectory of the students, sparse research has been carried out to empirically explore it [4,9]. In the Moldovan context, there is also limited research that investigates the importance of subject-specific mastery motivation for middle school students. Given the fact that school subjects differ in competences, skills, tasks, teachers, etc., the individual mastery motivation of the students in subject-specific contexts also varies, thus influencing academic outcomes specifically in middle school [9,10,17]. Therefore, the present study was conducted to address these research gaps by measuring subject-specific mastery motivation in middle schools and plausibly exploring the grade, gender, and language of instruction.

## 2. Theoretical Framework

### 2.1. Evolving Concept of Mastery Motivation

Mastery motivation is a multifaceted concept that is focused on the process of accomplishing an ongoing task regardless of the possible challenges [18]. The focal components of mastery motivation discriminating it from other concepts of motivation are the focus on the cognitive, object, or social persistence during the process of achieving/attempting to achieve a task in a specific domain along with the emotions that arise during the process of mastering endeavors [19,20,21]. Thus, mastery motivation is a process aimed to accomplish a task that is at least not easy and not the finally attained outcome. Mastery motivation theory is based on a two-aspect framework including the instrumental and affective aspects [19]. The instrumental aspect consists of four domains: cognitive/object persistence, gross motor persistence, social persistence with adults, and social persistence with children. The affective aspect refers to the positive or negative reaction of a person during the process of mastering a task or acquiring a skill materialized into mastery pleasure and negative reactions to challenge domains [22]. The multifaceted conceptualization of mastery motivation was first established in America and the concept has since evolved in many contexts of other countries such as Hungarian, Taiwanese, Iranian, and Moldovan school-aged students [23], Turkish, Chinese, Spanish, Bangla, and Iranian school contexts [24], Indonesian contexts [25], and Kenyan educational contexts [26].

### 2.2. Subject-Specific Mastery Motivation

In addition to the multifaced aspect of mastery motivation, there has been research regarding the domain specificity of mastery motivation [5]. As highlighted by Józsa et al. [27], the domain specificity of mastery motivation is scarce since this is a new area of research in mastery motivation. From a theoretical standpoint, additional dimensions of mastery motivation we described, assuming that mastery motivation is school-specific, assumes that mastery motivation level fluctuates in specific contexts. The recent trends in motivational research focus in motivational research has shifted from general domains to specific domains [28]. The rationale underlying the study of domain-specific mastery motivation points to the fact that motivational constructs in a general domain may or may not emerge or repeat the same trajectory/pattern in specific situations [7,29]. In addition, the skills and competences that are required in studying a specific subject differ, which can impact motivation perceptions across domains [30]. Specifically contextualized motivation measurements can increase the predictiveness of school achievement compared with the domain general motion instruments [9,31]. In predicting increased school achievement, stakeholders (e.g., parents, teachers, curriculum designers including textbook authors) play a key role when making decisions about the child’s mastery motivation [32]. Therefore, the role of the stakeholders is crucial to be able to help students’ learning processes adapt their subject-specific mastery motivation and mediation roles of certain subjects.

Thus, Józsa, proceeding from Barrett and Morgan’s definition of mastery motivation, designed a scale for measuring domain-specific dimensions of mastery motivation in the following subjects: reading, mathematics, science, English as a foreign language, German as a foreign language, arts, and music [33]. The measurement was developed on the foundation of the cognitive/object persistence domain. Thus, cognitive persistence precisely matters in challenging cognitive activities as performance is not conditioned just by the cognitive ability of a person but also by cognitive persistence, which is the motivation to achieve a task or performance as well as the effort an individual devotes to a task [34,35,36,37,38]. It is considered that cognitive persistence affects performance in challenging cognitive tasks that are at the person’s intermediate ability level and not the tasks that are higher or easier than the person’s ability level [37]. Józsa et al. [27] identified that students in primary can differentiate among their perception of mastery motivation in reading, mathematics, science, English/German as foreign language, art, and music. 

Thus far, very few studies have researched subject-specific motivation. Studies showed that elementary and secondary school students have differentiated mastery motivation in different academic domains [27,33]. Józsa found medium-cross domain correlations between the reading and math mastery motivation for fourth graders from Taiwan and Hungary. Moreover, based on their cross-sectional study, they concluded that when reading, mathematics, science, music, and arts decreased in both countries, English mastery motivation fluctuated. So, while in Hungary, it decreases minimally from grade four to six and remains stable from grade six to ten, it decreased constantly from grade four to eight, increasing in grade ten to the level of grade four in Taiwan [24]. In another study, when comparing the English mastery motivation with German mastery motivation, it was concluded that the level of German mastery motivation constantly decreased, whereas the English mastery motivation fluctuated [33]. These studies proved the contextual variation of mastery motivation and the need for further research.

Another issue that is worth investigating is the differential distinctiveness of the SSMMQ with age. Józsa et al. [27] discussed the correlations of the SSMMQ scales in the fourth and tenth grades finding a general declining tendency of the correlations. In contrast, Marsh and Ayotte (2003) proposed that with age and cognitive development of the children, the closely related areas of self-concept would be less differentiated and oppositely the divergent areas would be more differentiated, which should be visible in the correlations among the factors [39]. This process is in agreement with the concept of the Matthew effect that stands for the “rich-get-richer and the poor-get-poorer” trajectory [39,40]. Thus, we assume that the differential distinctiveness of the SSMMQ scales will be established for the disparate subjects resulting from decreasing paths of correlations among specific-subject mastery motivation scales across the fifth and the ninth grades.

### 2.3. Context of the Republic of Moldova

Historically, the Republic of Moldova has been a multicultural country where education is offered in two languages: Romanian and Russian. That is, in this country, there are monolingual schools (with either Romanian or Russian language of instruction) and mixed schools where there are classes that study in either Romanian or Russian. The language of instruction is chosen by the student’s parents or tutors. For this reason, all the educational materials used in the Moldovan pre-university system of education are issued in both Romanian and Russian. The linguistic and educational context of the Republic of Moldova allows the validation of measurement instruments in both the Romanian and Russian languages.

### 2.4. Current Study

To the best of our knowledge, no empirical study has addressed the role of subject-specific mastery motivation of secondary school students in the Republic of Moldova. Moreover, the studies on domain specificity of mastery motivation constructs employed exploratory factor analysis and correlations between subject-specific mastery motivation factors to study the mastery motivation constructs in the school context [27,33]. Therefore, this study aims to use structural equation modeling that will give further insight into the dimensionality of the SSMMQ. Another issue that has not been addressed in previous research is the comparison of latent mean differences across different groups, allowing a better understanding of the subject-specific motivation and the source of its variation. Thus, in the present study, we address the following research aims: (a) to explore the psychometric properties of the Romanian and Russian versions of the SSMMQ; (b) to analyze the age-related differential distinctiveness of the SSMMQ; (c) to explore different degrees of measurement invariance of the SSMMQ across language, grade, and gender; and (d) to investigate the latent mean differences across languages, grade levels, and gender and their magnitudes. Based on these research aims, we planned to address the following research questions.

RQ_1_: What are the psychometric properties of the Romanian and Russian versions of the SSMMQ?
RQ_2_: Is there an age-related (grade levels) differential distinctiveness in the SSMMQ?
RQ_3_: What is the measurement invariance of the SSMMQ across language, grade levels, and gender?
RQ_4_: What are the latent mean differences across languages, grade levels, and gender?

## 3. Method

### 3.1. Participants

We employed stratified sampling which included the following explicit stratification variables: (a) schools that offer instruction in just one language, monolingual schools, and mixed-language schools; (b) schools that teach English as the first foreign language; and (c) regarding language of instruction, schools with Romanian language of instruction and schools with Russian language of instruction. All these schools contain at least all of the following levels: ISCED 1 (primary school), ISCED 2 (middle school/gymnasium), and ISCED 3 (high school/lyceum). Therefore, the one implicit stratification variable was schools that offer ISCED 2 level. The total number of the schools that participated in this study was five: two schools with Russian and three with Romanian language of instruction. Since ISCED 2 in the Republic of Moldova comprises grades five to nine, and to meet the objective of determining the trajectory of SSMM in middle school, we selected the entry grade (fifth), the exit grade (ninth), and the one in the middle (seventh).

The sample comprises 939 (472 girls and 466 boys) secondary school students from five public schools in a large city in the Republic of Moldova. The response rate within schools in this study was 90.70%. Two linguistically different samples were used: the Romanian (RO) sample consisting of the students who studied in schools with the Romanian language of instruction (*N*_RO_ = 586 (62.407%)) and the Russian (RU) sample corresponding to the students studying in schools with the Russian language of instruction (*N*_RU_ = 353 (37.593%)). Moreover, the distribution across grade levels was the following: 346 (36.848%) with an average age of 11.147 (*SD* = 0.436) studied in the fifth grade (*N*_RO, 5_ = 219 and *N*_RU, 5_ = 127), 304 (32.375%) aged 13.059 (*SD* = 0.410) studied in the seventh grade (*N*_RO_, _7_ = 199 and *N*_RU, 7_ = 105 Russian), and 289 (30.777%) aged 13.051 (*SD* = 0.326) were in the ninth grade (*N*_RO, 9_ = 168 and *N*_RU, 9_ = 121). 

### 3.2. Instrument

In the current study, the Subject Specific Mastery Motivation Questionnaire (SSMMQ) [27,33] was used. The Subject-Specific Mastery Motivation Questionnaire (SSMMQ) contains the following scales: Reading Mastery Motivation (Reading), Mathematics Mastery Motivation (Math), Science Mastery Motivation (Science), Music Mastery Motivation (Music), Art Mastery Motivation (Art), English as a Foreign Language Mastery Motivation (English), German as a Foreign Language (German), and School Mastery Pleasure (SMP). The English and German scales are used depending on the foreign language that is studied by the student. Each scale consists of six 5-point Likert items. The scales do not consist of parallel items. The SMP scale consists of six items that are worded in parallel, i.e., they all begin with the same words: ‘I am pleased when…’. In the present study, we used the Reading, Math, Science, English, Art, Music, and SMP scales. Previous psychometric studies of the SSMMQ supported a seven-factor structure. The Cronbach’s alphas of the Hungarian version of the SSMMQ ranged from 0.785 to 0.923: the SMP had the lowest internal consistency and Music scale had the highest one. The reliability of the Taiwanese version ranged from 0.786 for SMP to 0.915 for English [27]. 

All SSMMQ scales are reflected in the subjects that are taught in middle school in the Republic of Moldova, e.g., Reading is taught in the Romanian/Russian language and literature, Math in mathematics, Science is taught in the fifth grade as science, from the sixth to ninth grades, physics is taught, and from the seventh to ninth grades, chemistry is taught. Foreign Language is in the students’ curriculum throughout middle school. Music and Art are practiced within music education, art education, and technological education. 

### 3.3. Translation of the SSMMQ English into Romanian and Russian

Having received permission from the author of the SSMMQ, the questionnaire was translated into Romanian and Russian [41]. Due to the fact that the SSMMQ was translated and validated simultaneously in Russian and Romanian in the same context, we used bilingual expert translators (Romanian and Russian). The stages used in this translation process were: (a) forward-translation done by two translators, (b) forward-reconciliation and harmonization among versions, (c) back-translation, (d) evaluation of the back-translation by an expert in mastery motivation, (e) pilot testing of the Romanian and Russian versions of the SSMMQ on fifth-grade students, and (f) final review based on pilot results [42]. 

### 3.4. Data Collection

During the preparation stage of data collection, the researchers sought ethical approval from the Institutional Research Board of the University of Szeged and the research followed all procedures requested by the educational institutions where the data were collected. The questionnaire was filled in during class hours using the pen and paper method. Each session lasted 45 min. One of the researchers administrated the questionnaire across all the sessions, ensuring consistent procedures for all questionnaire takers. There was no student who refused to participate. Having completed the questionnaire, students were encouraged to give some feedback and ask questions related to the SSMMQ.

### 3.5. Analytical Procedure

The procedure used in the study of the equivalence in the latent structure of subject-specific mastery motivation among groups was sequential and progressive. The first step was to analyze the pattern of the missing data and multiply impute the data using SPSS 28.0. Next, normality tests (skewness and kurtosis) were carried out to determine if the data fit the normal distribution. 

To establish the dimensionality and psychometric properties of the Romanian and Russian versions of the SSMMQ, we first conducted confirmatory factor analysis (CFA) to analyze the factor structure of the SSMMQ using the overall sample. Thus, we assessed the following models: (1) Model 1 reflected the original seven-factor Subject-Specific Mastery Motivation model proposed by Józsa et al. [27] that included six subject-specific factors (Reading, Math, Science, English Art, and Music) and one school mastery pleasure factor; each factor consisted of six items; (2) Model 2 was a six-factor model in which the school mastery pleasure variables were included in the subject-specific factor, thus each factor contained seven items; and (3) Model 3 contained only six subject-specific factors, with six-items per factor. To ascertain the adequacy of model fit measures of the model, we examined the comparative fit index (CFI), Tucker–Lewis index (TLI), root-mean-square error of approximation (RMSEA), and standardized root mean squared residual (SRMR) [43]. The chi-square test was used to select the best structural models among the nested models. However, due to both the sensitivity of the chi-square test to sample size and statistically significant chi-square results produced in large samples, we considered other fit indexes, namely CFI, TLI, RMSEA, and SRMR. CFI and TLI have values that are highly correlated. Some researchers recommend that one of them be reported even though TLI has a greater relative penalty for model complexity [44]. Moreover, there are studies that concluded that TLI was the only index that had values that were not dependent on sample size [45]. SRMR was used for assessing model fit as it is more accurate when there are a large number of variables in comparison with RMSEA [46]. To evaluate the fit indexes, we referred to the following cut-offs: TLI and CFI ≥ 0.95, RMSEA ≤ 0.06, and SRMR ≤ 0.08 [47,48]. 

Next, reliability, mean, and standard deviation values were computed. The reliability of the Romanian and Russian versions of the SSMMQ was estimated using internal consistency reliability by calculating Cronbach’s alpha, and composite reliability (CR) was measured by coefficient omega (ω) [49]. Values above 0.700 are considered acceptable [50]. To evaluate the construct validity of the investigated version of the SSMMQ, we studied its convergent and discriminant validity. Convergent validity was proved if the factor loadings were above 0.400, with a sample size of 400 for significance, if CR was higher than 0.700, and average variance extracted (AVE) exceeded 0.500 [46]. AVE values that did not meet the 0.500 cut-off could be accepted if the CR of the factor was above 0.60 [51]. Discriminant validity was evaluated by a more traditional approach, the Fornell–Larcker criterion, which has low sensitivity, and by a more modern approach, the heterotrait-monotrait ratio of correlations (HTMT). For the Fornell–Larcker criterion, AVE values should be higher than the squared inter construct correlation estimate. The threshold for HTMT is 0.850, which is quite a conservative one and suggests that the constructs are more distinct [52]. 

Next, to investigate the validity of the SSMMQ and factor latent differences, we determined the measurement invariance (MI) (by the language of instruction of the student, grade level, and gender). Thus, we identified a model that would fit all the groups, and the baseline model for each group was separately analyzed using CFA with maximum likelihood estimation. MI substantiates the equality of factor pattern on the latent factor (configural invariance), the equality of factor loadings (metric invariance), the equality of item intercepts (scalar invariance) confirming comparisons of factor means across groups, and the equality of item residuals of metric and scalar invariant items (residual invariance) [53]. Full scalar invariance is a desideratum that is difficult to achieve especially on a cross-cultural level; therefore, in case of lack of full invariance, partial invariance could be established [54]. Residual invariance is not a compulsory step for demonstrating full factorial invariance, but it is not required for the interpretation of latent means [55]. The invariance was accepted with a ΔCFI ≤ 0.010, ΔTLI ≤ 0.010, and ΔRMSEA ≤ 0.015, in favor of the least strict model [56]. 

We opted for using the test of latent means differences to compare the groups. This test does not provide an increase in statistical power in comparison with the traditional multivariate analysis (e.g., *t*-test, ANOVA) [57]. Thus, to determine the model, the latent mean of one group was constrained to zero (reference group) while the latent mean of the second group was freely estimated (comparison group) [58]. Thus, to calculate the latent mean difference across grade levels, we first constrained the fifth-grade latent mean to zero, and the other two group means were freely estimated to obtain the mean differences between the fifth and seventh graders and the fifth and ninth graders. Next, the latent mean of the seventh grade was set to zero, and the other two groups were freely estimated to generate the latent mean difference between the seventh and ninth grades [59]. For language and gender, the reference groups were female and Russian.

The critical ratio (CR) index was used to evaluate the estimated latent mean difference across groups. The CR represents parameter estimate divided by its standard error and it is used to determine if the estimate is statistically different from zero [60]. CR values exceeding ±1.96, which corresponds to a 0.05 error level, are considered significant [50], and a negative CR value indicates that the comparison group has lower latent mean values than the reference group. However, in the 5-point Likert scale used in the SSMMQ, there is no true zero value. Therefore, we used Cohen’s d as effect size index to interpret the mean differences in terms of their magnitude [53,61]. The data were analyzed using IBM SPSS and IBM SPSS AMOS (Version 28.0).

### 3.6. Preliminary Data Analysis

The initial sample consisted of 942 students. Three students had more than 30% of missing data and were omitted from the study. Before carrying on any statistical analysis, we investigated the frequency of the missing data, its randomness, and patterns. Thus, 9.265% of the subjects contained incomplete data. Missing data constituted 0.400–2.200% on individual items due to non-response, which is in agreement with Kline’s recommendations for treating the issue of missing values across items and cases [44]. 

Two tests were carried out to explore the degree of randomness in the missing data. First, Little’s Missing Completely at Random test revealed that the missing pattern could not be considered to be missing completely at random (χ^2^ = 2054.689, *df* = 1622, *p* < 0.001) [62]. The next step was to determine the propensity for data points, i.e., if the missing values were associated with some of the observed variables [63]. A series of t-tests revealed that the missing values across some factors were related to the values of other observed factors. Therefore, we concluded that the missing value pattern was Missing at Random (MAR). According to Graham’s [64] recommendations, the multiple imputation (MI) approach was selected to deal with missing values. 

The means and standard deviations of the 42 variables are presented in Table 1. Since the maximum-likelihood method was used in the factor analysis, we investigated the normality of our data [65]. Curran et al. (1996) proposed that the normal distribution should not be severely violated [66]. Kline’s (2016) guideline of severe non-normality indicated that a severe violation of normality assumption was defined by skewness (Sk) values which are greater than 3 and kurtosis (K) values which are greater than 10 [44]. As a result, the skewness values of the Romanian sample were between −1.613 and 0.221 and kurtosis scores ranged from −1.387 and 1.818, while the skewness values of the Russian sample were between −1.814 and 0.266 and kurtosis scores ranged from 2.765 and −1.382. These values suggest that all variables showed relatively normally distribution.

## 4. Results

### 4.1. Dimensionality of Romanian and Russian Versions of SSMMQ

First, we tested the seven-factor SSMMQ model; each factor contains six items (Model 1) proposed by Józsa et al. (2017) that included Reading, Mathematics, Science, English, Music, and Art Mastery Motivation, and School Mastery Pleasure scales [27]. As presented in Table 2, Model 1 did not lead to a sufficient model fit in either the Romanian (χ^2^ (798, *N* = 586) = 2605.054, *p* < 0.001, CFI = 0.897, TLI = 0.889, RMSEA = 0.062 [0.060, 0.065], SRMR = 0.064) or Russian samples (χ^2^ (798, *N* = 352) = 1998.593, *p* < 0.001, CFI = 0.879, TLI = 0.869, RMSEA = 0.065 [0.062, 0.069], SRMR = 0.070). All standardized factor loadings for this model ranged from 0.581 to 0.933 in the Romanian version; the lowest factor loadings had SMP scale (0.581–0.685). In the Russian version, the factor loadings were lower than the Romanian factor loadings, ranging from 0.531 to 0.923, and again in the Russian version of SMP, the lowest factor loading was yielded by the SMP scale (0.531–0.652). 

Model 2 contained the same number of variables, but the SMP items were included in the respective subject-specific mastery motivation scale; thus, it contained six dimensions and 42 items. It had an improved model fit for the Romanian version (χ^2^ (804, *N* = 586) = 2255.256, *p* < 0.001, CFI = 0.917, TLI = 0.912, RMSEA = 0.056 [0.053, 0.058], SRMR = 0.056) as well as for the Russian version (χ^2^ (804, *N* = 352) = 1780.355, *p* < 0.001, CFI = 0.901, TLI = 0.894, RMSEA = 0.059 [0.055, 0.062], SRMR = 0.061). The factor loadings of the 42 SSMMQ items in the Romanian version ranged from 0.476 to 0.930. The items of the SMP dimension had the lowest factor loadings, proving that they do measure a different factor (0.476–0.611). The Russian version of the SSMMQ with factor loadings of 0.456–0.922 followed the Romanian pattern and the items representing SMP loaded the lowest on the subject-specific scales (0.456–0.696). The model fit of the SSMMQ considerably improved with dropping the SMP scale.

As a result, Model 3 (in which the six variables measuring SMP were deleted, thus it included six dimensions comprising six items each and no correlations were imposed) had a good model fit when compared with the other two models. The Romanian yielded the following fit indexes: χ^2^ (579, *N* = 586) = 1433.384, *p* < 0.001, CFI = 0.944, TLI = 0.940, RMSEA = 0.050 [0.047, 0.054], SRMR = 0.041, whereas that of the Russian version was as follows: χ^2^ (579, *N* = 352) = 1228.756, *p* < 0.001, CFI = 0.926, TLI = 0.919, RMSEA = 0.056 [0.052, 0.061], SRMR = 0.050. These results indicated that model fit did not meet the standard criteria of good fit in both versions of the SSMMQ. Having studied the results of modification indices, we gradually co-varied error terms in each version individually. Thus, χ^2^ values decreased. Then, we added five covariances of item errors producing model fit values that met the thresholds. In the Romanian version, the imposed covariances were between the residuals of the following items: Music 1 and Music 2, Music 3 and music 6, English 1 and 2, Art 6 and 5, and Reading 6 and Reading 4. The final SSMMQ model of the Romanian version showed that all the goodness-of-fit indices met the fitting criterion: χ^2^ (579, *N* = 586) = 1090.799, *p* < 0.001, CFI = 0.966, TLI = 0.963, RMSEA = 0.039 [0.036, 0.043], SRMR = 0.041 (Table 2), with factor loadings ranging from 0.618 to 0.947 (Table 3). In the Russian version, five covariances were Music 1 and Music 2, English 1 and 2, Art 2 and 4, Reading 1 and Reading 4, and Reading 4 and 6. The fit indexes of the final modified model of the Russian version were acceptable: χ^2^ (579, *N* = 352) = 985.752, *p* < 0.001, CFI = 0.953, TLI = 0.948, RMSEA = 0.045 [0.040, 0.050], SRMR = 0.050 (Table 2); the factor loadings in this model were between 0.560–0.932 (Table 3).

A concern of this study is the possible ceiling effect in English scales (Table 1). Approximately, 21.299% of the students endorsed the highest option of the Likert scale, which is above the cut-off of 20% [67]. We identified a ceiling effect of 24.403% in the Romanian sample and 16.147% in the Russian sample. Next, we studied the floor effect in the Music scale as it yielded the lowest scores. A floor effect of 13.418% was established for the whole sample. The Romanian sample produces a floor effect of 11.433%, whereas the Russian one was 16.714%; thus, as these numbers are below the acceptable value of 20%, the floor effect in the Music scales was not identified.

### 4.2. Validity

In terms of convergent validity, AVE values of the Romanian and Russian versions of the SSMMQ were assessed as demonstrated in Table 3. All AVE values for the Romanian sample were above 0.500, whereas the AVE of the Russian version for the Reading scale is 0.497 (which is below the threshold of 0.500); however, the CR is 0.854 (which exceeded the cut-off point of 0.060 and allowed us to accept this AVE value).

To further examine the discriminant validity of the SSMMQ, the heterotrait-monotrait ratio of correlations (HTMT) analysis was carried out. Table 4 shows that all HTMT values for both versions were <0.85, establishing the discriminant validity for the Romanian and Russian six-factor models. The discriminant validity of the SSMMQ was also estimated using Fornell and Larcker’s approach. The square root of AVE of each latent factor of the SSMMQ was higher than the correlations it has with the rest of the latent variables in the model in both samples, as presented in Table 5.

The relationship between factors of the SSMMQ was analyzed by language and grade level. The correlations among scales by language varied from low to moderate positive: for the Romanian sample, *rs* = 0.167 to 0.520 and median = 0.391, and for the Russian one, *rs* = 0.154 to 0.486 and median = 0.297. The lowest correlations in both samples emerged for English and Art (*r*_RO_ = 0.167, *r*_RU_ = 0.157), English and Music (*r*_RO_ = 0.187, *r*_RU_ = 0.243), and Math and Art (*r*_RO_ = 0.191, *r*_RU_ = 0.174) (Table 6). Regarding the correlations by language grade, the significant fifth-grade correlation among the SSMM scales varied from 0.131 to 0.532; median = 0.375, and the correlation between English and art was not statistically significant. All the correlations for the seventh grade were significant (*rs* = 0.201–0.503; median = 0.383). The ninth-grade correlations that were statistically significant ranged between 0.151 and 0.488; median = 0.347, but the Math scale was not statistically significantly related to the Music and Art scales. The correlations across the Reading, Science, and Math scales had a constantly declining trajectory from fifth to ninth grades. Some of the correlations had an increasing path, namely between Reading and English, Reading and Art, Science and English, Science and Music, and English and Art.

### 4.3. Measurement Invariance of the SSMMQ

#### 4.3.1. Baseline Model

To establish the comparison standard for latent means comparison across different groups first, a baseline model was established. Initially the original seven-factor SSMMQ model was assessed independently in each group (language, grade level, and gender). In this model, no measurement errors were correlated. The results of this analysis are presented in Table 7.

The next step was to analyze the modification indexes of each sample separately, allowing measurement errors to be correlated [58]. The main purpose of this stage was to determine a baseline model that would fit all the groups (language, grade, and gender) and that would allow us to establish measurement invariance. Therefore, we first calculated the fit indices for the model with correlated errors in each sample, as shown in Table 7. The fit values met the thresholds in all the groups except for the Russian and ninth grade groups. The second stage was to identify the correlation that was present in all the samples which led to a strong baseline model that could be replicable in all samples and which would avoid accidental augmentation of fit indexes. Therefore, the final model used for measuring invariance was modified by establishing the residual covariation correlation between the two items Reading 4—Reading 6, Art 6—Art 5, English 2—English 1, English 4—English 2, and Music 1—Music 2. After re-specification, the baseline models that included the same error correlations were estimated. The fit values were lower in some groups in comparison with the previous model. These results revealed acceptable model fit to the data. This last model was used for testing measurement invariance.

#### 4.3.2. Invariance across Languages

The configural model was evaluated and it produced a good baseline model fit for all indexes (Table 8). In assessing the metric invariance, the factor loadings were constrained to be equal across Romanian and Russian students. Comparison of configural and metric models did not show any decrease in fit, i.e., the factor loadings were fully invariant across languages (ΔCFI = −0.006, ΔRMSEA = 0.001, ΔSRMR = 0). To test for the scalar invariance, the intercepts of all items were constrained to be the same across the groups. However, the change between metric and scalar invariance indicated that the intercepts were not equal based on the ΔCFA (ΔCFI = −0.011, ΔRMSEA = 0.002, ΔSRMR = 0.004). In pursuit of the partial scalar invariance model, we unconstrained each intercept to establish where the misfit between the Romanian and Russian groups occurred (Vandenberg & Lance, 2000). The results revealed that item Music 4 was the cause in the change in CFI. By letting this intercept free, no significant change was occurred in the fit between the metric and the partial scalar model (ΔCFI = −0.009, ΔRMSEA = 0.002, ΔSRMR = 0.004). By constraining item residuals in the partial scalar model, the residual invariance was tested. The fit indexes supported this residual model as well (ΔCFI = −0.010, ΔRMSEA = 0.003, ΔSRMR = −0.003). These results revealed that the intercepts and residual variances were partially invariant across languages [56].

#### 4.3.3. Invariance across Grades

The configural model was tested and demonstrated good model fit to the data (χ^2^ (1719) = 2887.185, CFI = 0.951, RMSEA = 0.028 [0.026, 0.029], SRMR = 0.051). The difference in examined criteria between the model with equal factor loadings and the configural did not suggest a decrease in fit (ΔCFI = −0.001, ΔRMSEA = −0.002, ΔSRMR = −0.004). Given this, we proceeded to measuring the scalar invariance, which also yielded a small decrease in the fit (ΔCFI = 0.008, ΔRMSEA = 0.002, ΔSRMR = 0.001). As presented in Table 8, the comparison of the scalar versus residual invariance models did not suggest a meaningful decrease in fit (ΔCFI = 0.003, ΔRMSEA = 0, ΔSRMR = 0). Given this empirical evidence, configural, metric, and scalar and residual invariance for the six-factor SSMMQ was demonstrated.

#### 4.3.4. Invariance across Gender

The configural model was assessed to establish if it was a good representation of the hypothesized relationships in the SSMMQ across gender. The results suggested evidence for a good model fit (χ^2^ (1146) = 2085.271, CFI = 0.959, RMSEA = 0.030 [0.028, 0.032], SRMR = 0.041). The comparison between the configural and metric models showed a change in all studied indexes that met the cut-points (ΔCFI = −0.001, ΔRMSEA = 0, ΔSRMR = 0.001). The decrease in fit between the metric and scalar was insignificant (ΔCFI = 0.009, ΔRMSEA = 0.002, ΔSRMR = 0.002). Comparing the residual invariance model against the scalar invariance model, we did not identify a decrease in fit indexes (ΔCFI = −0.003, ΔRMSEA = 0, ΔSRMR = 0.001), as shown in Table 8.

### 4.4. Latent Mean Differences

Upon the establishment of full scalar invariance across gender and grade, and partial scalar invariance across languages, the latent means differences can be compared. In this analysis, the Russian and female groups were used as reference groups for languages and genders. However, when comparing the fifth vs. seventh and fifth vs. ninth grades, the fifth grade was constrained to zero, and when comparing the seventh and ninth grades, the seventh grade was defined as the reference group (Table 9).

The study of the latent mean differences by language demonstrated that the Russian group had lower means than the Romanian one on the Music, Math, and Science scales but the effect size of these mean differences was small, which can be neglected. The analysis of latent mean differences of the SSMMQ scales across gender showed that females had higher scores than males in all scales except for Math. The mean differences in Music, Art, and Reading had a medium effect size, whereas the rest had a small one. When comparing the means between the fifth and seventh grades, we found that these grades differed on Music, Art, and Math scales, the fifth grade having higher means with a small, even negligible, effect size, as shown in Table 9. Significant mean differences between the seventh and ninth grades were estimated in Art and English. The results of the latent mean differences between the fifth and the ninth grades revealed that the ninth graders reported lower levels of Music, Art, English, Math, and Science but with small effect size.

## 5. Discussion

The current study aimed to explore the factor structure of the Romanian and Russian versions of the SSMMQ in a sample of fifth, seventh, and ninth graders from the Republic of Moldova. For this purpose, we tested three models of the SSMMQ: the first one was the initial seven-factor model of the SSMMQ that was put forward by Józsa et al. [27]. It did not produce an acceptable fitness of good in both versions. Given the fact that the school mastery pleasure items had the lowest factor loadings and that each of its items measured the mastery pleasure in the specific subjects comprised in the SSMMQ, we included these items in the subject-specific scales for being the second tested model. The last model of the SSMMQ in Romanian and Russian included only the six subject-specific scales (six items per scale) and all the items assessing school mastery pleasure were excluded; it yielded the best goodness of fit indices and good internal consistency values across all samples.

The variables of school-specific mastery pleasure in the original study cross-loaded above 0.400 on the corresponding subject-specific mastery scale (English, Science, Art, and Music) and school-specific mastery pleasure scale [24]. We consider that an item of SMP can be dropped only if the whole related school domain scale is dropped. Otherwise, the drop of an SMP item related to a scale used in the questionnaire violates the construction of the construct of subject-specific mastery motivation. SMP is an affective scale that measures the expressive aspect during or right after mastering subject-specific tasks, which is similar to the Mastery Pleasure scale in the Dimension of Mastery Questionnaire 18 (DMQ 18). The items assessing mastery pleasure in the DMQ 18 are worded diversely while the items evaluating school subject mastery motivation in the SSMMQ are worded in parallel. Thus, in the DMQ 18, mastery pleasure is worded by a variety of phrases, e.g., “I smile when …”, “I get excited when …”, and “I am pleased …”, whereas in the SSMMQ, each item starts with “I am pleased when …”. Parallel wording in scales can cause misfits or inadequate fit and biased outcomes [68]. To our knowledge, there is no statistical solution for scales that are composed of items with parallel wording. We hypothesize that the subject-specific mastery pleasure items would be varied to express “smiling, laughing or other behavioral indicators of positive affect” during or after mastering tasks in the evaluated subjects.

One of the issues that we identified in this study is the presence of a ceiling effect in the English as a foreign language mastery pleasure scale. A ceiling effect occurs when the participants select the highest option on the Likert’s scales, thus hampering the possibility of measuring the true extent of their subject-specific mastery motivation in our case. A ceiling effect can be a source of bias and it can limit the instrument’s potential for differentiation among participants [68]. This is the first time a ceiling effect was discussed on the English scale of the SSMMQ [27,33].

Evidence for sufficient internal consistency was indicated by Cronbach’s alpha and CR, which exceeded 0.700 in both the Romanian and Russian versions. One issue of some concern regarding the internal consistency is the alpha values of the Music, Art, and English scales in the Romanian version of the SSMMQ and Music and Art scales in the Russian version. The acceptable values of coefficient alpha range from 0.600 to 0.950 [66]. Nevertheless, there are some researchers who consider that values above 0.900 may point to a possible content overlap across items [69]. In the original research, higher values of coefficient alpha were computed in the Music scale in the Hungarian and Taiwanese samples and the English scale in the Taiwanese sample [24]. The results of the psychometric analyses exhibited adequate construct validity of the SSMMQ. The various psychometric analyses showed evidence of the convergent and discriminant validity of the Romanian and Russian versions of the SSMMQ. These findings provide initial psychometric evidence for the validity of the SSMMQ in the context of the Moldovan educational system where education is provided in the Romanian or Russian languages.

Another question that was investigated in this study is the differential distinctiveness of subject-specific mastery motivation by students of different grades (age levels). Following Marsh and Ayotte’s (2003) train of thought, we assumed that there would be a declining trajectory of the correlations among mastery motivation factors in divergent subjects [39]. We identified that there was a systemic decrease of correlations among Reading, Math, and Science scales from the fifth to the ninth grade, which is in agreement with Józsa et al. [27] especially for the Taiwanese sample. This decreasing path suggested that the ninth-grade students perceived these scales as more distinctive, which can be explained by the fact that they have increased cognitive development and a more extensive academic experience, allowing them to better differentiate these subject domains. The rest of the correlations have either a negligible increasing or decreasing trajectory, which is in disagreement with a previous study. This path could be explained by the fact that some of the subjects included in the SSMMQ have common competences, for instance, Reading and English. These finding are congruent with Marsh and Ayotte [39] who constructed the differential distinctiveness hypothesis that stated that as children grew older, they were more likely to differentiate factors that are theoretically more distinctive. Nevertheless, there is a need for further analysis of differential distinctiveness of the SSMMQ, as the changes of the fifth and seventh grade and between the seventh and ninth grades have a different trajectory from those identified between the fifth and ninth graders.

The complexity of this study resides in the inclusion of three criteria in defining groups (language (Romanian and Russian), grade (five, seven and nine) and gender), resulting in the use of seven different groups in the statistical analysis. This complexity motivated the adoption of a sequential approach to defining the baseline model for further measurement invariance. The correlated errors imposed on the final baseline model were selected on the criteria of being present in all the groups to avoid accidental deflation or inflation of statistical outcomes.

On measurement invariance, the results supported the pattern structure, the factor loading, the item intercept, and the item residual variance across language, grade, and gender. The only partial scalar invariance that was established was across the ethnic groups where one intercept was freed. Partial scalar invariance points to the fact that a group can interpret the distances between points on the Likert scale shorter or longer on a particular item in comparison with the other groups [61]. The potential causes of these individual ethnic or cultural interpretations can be the propensity of a group to adhere to some social norms, the use of different criteria when evaluating themselves, or the overrating of a value or trait that is considered a weakness in their culture [56,70]. Importantly, this finding does not affect the validity and reliability of the Romanian and Russian versions of the SSMMQ that were fully demonstrated and discussed above.

In this study, we also aimed to assess group-level differences in subject-specific mastery motivation. In the studied sample, the means of girls were higher than those of boys. Thus, there is a statistically significant difference in means in Science and English mastery motivation, whereas in Art, Music, and Reading, the difference was a medium. There was no gender difference in the level of Math mastery motivation. Gender differences have rarely been examined within the theory of mastery motivation. The only study that focused on these differences used the Dimensions of Adult Mastery Motivation Questionnaire that investigated mastery motivation levels in university students [4]. This study found that there was a lack of gender differences in Hungarian students, but the Australian, Bangladeshi, and Iranian female students reported significantly lower levels of mastery motivation.

What is more, the lack of gender differences in Math mastery motivation is in disagreement with studies that investigated motivation at school and concluded that secondary school girls (as compared with boys) have lower mastery motivation in Western countries [71]. At the same time, there are several studies that have identified that boys reported lower academic or domain-specific motivation than girls in Belgium, Russia, Azerbaijan, Australia, and the US [72,73,74,75]. In light of the new emergent gender roles, the gender differences in subject-specific mastery motivation can explain the academic fluctuations of the students. Nevertheless, the gender differences may be age- or grade- related as, at the university level, there are no differences between males and females on the total mastery motivation and on the scales of Dimensions of Adult Mastery Motivation Questionnaire College [76].

Although the students studying in the Romanian language had higher latent means of Music, Science, Art, and Science mastery motivation, the size effect of these differences are below 0.200, therefore they are negligible. Thus, there was no statistically significant difference between the latent means of the students receiving education in the Romanian language and those studying in Russian.

Findings also showed latent mean difference across seventh graders had a lower Music, Art, and Math mastery motivation in comparison with fifth graders. Moreover, the ninth graders exhibited statistically and significantly lower mastery motivation in Art and English, whereas the latent mean comparison of fifth- and ninth-grade students revealed more differences, namely in Music, Art, English, Math, and Science mastery motivation, with the ninth graders having lower latent means. All identified latent differences had a small effect. One subject-specific mastery motivation level that remained stable across the grades was Reading. Art mastery motivation constantly decreased across the grades. English is the subject-specific mastery motivation that starts decreasing more significantly in the seventh grade, continuing towards the ninth grade. Music, Math, and Science mastery motivation decrease gradually but it is identified only in ninth graders and not in seventh graders. Some of the grade level changes found in this study correspond with the previous studies examining subject mastery motivation in Hungary and Taiwan. The Art, Science, and Math mastery motivation of the students from Hungary are similar with the ones from the Republic of Moldova and decreased across the grades with a similarly small effect size. English as a foreign language did not decrease in either Hungary or Taiwan at the secondary school level, whereas it did in Moldova, just like mastery motivation in all other subjects under investigation. Only in the Republic of Moldova was Reading mastery motivation level stable across the grades, which is opposite to the findings of the previous research. In Hungary, the English mastery motivation level tends to drop from the fourth to the sixth grade, but later on it becomes stabilized. Furthermore, the outcomes of the current study support the conclusions that the cognitive persistence domain of mastery motivation tends to decline in students from grade four to grade eight [5].

## 6. Limitations and Future Directions

The present study has several limitations. One of them is the cross-sectional design adopted for investigating the subject-specific mastery motivation across grades. A longitudinal study can reflect the students’ true personal changes over time. A further direction in the research on subject-specific motivation would be the analysis of the degree of independence of development of its constructs over time, its predictive power, and further development of the school-specific mastery pleasure domain.

## 7. Conclusions

The present study contributes to the empirical literature of subject-specific mastery motivation by translating the SSMMQ into Romanian and Russian and validating them in the context of the Republic of Moldova. As a point of psychometric properties measurement, the last SSMMQ model of both the Romanian and Russian versions was well-fitted after excluding all items assessing students’ school mastery pleasure, and it could prove that the SSMMQ was reliable and valid for measuring the subject-specific mastery motivation in Moldovan middle-school students. It was also identified that the SSMMQ of the students studying in the Romanian language does not differ significantly from the students having Russian as the language of instruction. Investigating age-related (grade levels) differential distinctiveness of the SSMMQ, a systematic decrease of correlation was found among the scales of Reading, Math, and Science from the fifth to the ninth grades. This decreasing correlation means that ninth graders are more distinctive in these subjects due to their increased cognitive development and extended academic experience. In the measurement invariance of the SSMMQ across language, grade, and gender, our study could demonstrate the residual measurement invariance across language, grade level, and gender. In addition, we identified that gender differences in the SSMMQ were significant, especially in Reading, Music, and Art; boys were less motivated to master a skill in these domains. Comparing the latent mean difference also gave a first insight into the domain-specific mastery motivation, showing no significant difference between the Romanian and Russian samples (with very low effect sizes across languages, grade levels, and genders).

## Figures and Tables

**Table 1:** Descriptive statistics: means and standard deviations.

Samples	Reading	Math	Music	Science	English	Art
Overall sample (*N* = 939)	3.651(0.846)	3.814 (0.878)	2.716 (1.310)	3.224 (0.952)	4.091 (0.912)	3.311 (1.266)
Romanian (*N* = 586)	3.691 (0.838)	3.879 (0.846)	2.802 (1.329)	3.270 (0.879)	4.128 (0.938)	3.304 (1.263)
Russian (*N* = 353)	3.584 (0.856)	3.708 (0.919)	2.573 (1.266)	3.148 (1.058)	4.029 (0.863)	3.321 (1.271)
5th grade (*N* = 346)	3.657 (0.833)	3.956 (0.824)	2.926 (1.290)	3.325 (0.961)	4.133 (0.873)	3.677 (1.126)
7th grade (*N* = 304)	3.652 (0.855)	3.675 (0.887)	2.629 (1.307)	3.222 (0.943)	4.183 (0.875)	3.231 (1.237)
9th grade (*N* = 289)	3.641 (0.856)	3.792 (0.907)	2.557 (1.308)	3.106 (0.940)	3.943 (0.978)	2.957 (1.340)
Female (*N* = 472)	3.867 (0.805)	3.859 (0.884)	3.023 (1.315)	3.363 (0.948)	4.241 (0.868)	3.718 (1.145)
Male (*N* = 466)	3.433 (0.832)	3.770 (0.871)	2.402 (1.230)	3.085 (0.937)	3.941 (0.930)	2.899 (1.251)

**Table 2:** Goodness of fit indicators of the models of the SSMMQ using confirmatory factor analysis.

Version	Model	χ^2^ (df)	χ^2^/df	TLI	CFI	RMSEA [90% CI]	SRMR
Ro	Model 1	2605.054 (798)	3.264	0.889	0.897	0.062 [0.060, 0.065]	0.064
	Model 2	2255.256 (804)	2.805	0.912	0.917	0.056 [0.053, 0.058]	0.056
	Model 3	1433.384 (579)	2.476	0.940	0.944	0.050 [0.047, 0.054]	0.041
	Model 3a	1090.799 (574)	1.900	0.963	0.966	0.039 [0.036, 0.043]	0.041
Ru	Model 1	1998.593 (798)	2.505	0.869	0.879	0.065 [0.062, 0.069]	0.070
	Model 2	1780.355 (804)	2.214	0.894	0.901	0.059 [0.055, 0.062]	0.061
	Model 3	1228.756 (579)	2.122	0.919	0.926	0.056 [0.052, 0.061]	0.050
	Model 3a	985.752 (574)	1.717	0.948	0.953	0.045 [0.040, 0.050]	0.050

*Note:* χ^2^ = chi-square; *df* = degrees of freedom; TLI = Tucker–Lewis index, CFI = comparative fit index; RMSEA = root mean square error of approximation; SRMR = standardized root mean squared residual; Model 1 = seven-factor model with 42 variables; Model 2 = six factor model with 42 variables; Model 3 = six-factor model with 36 variables; Model 3a = Model 3 with correlated errors.

**Table 3:** Factor Loadings, Composite Reliability, AVE, and Cronbach’s Alphas for the Romanian and Russian versions of the SSMMQ.

Items	Factor Loadings		Composite Reliability ω		AVE		Cronbach’s α	
	RO	RU	RO	RU	RO	RU	RO	RU
Music Mastery Motivation								
Music 3	0.899	0.873	0.949	0.932	0.757	0.697	0.952	0.936
Music 4	0.947	0.835						
Music 2	0.855	0.825						
Music 6	0.820	0.874						
Music1	0.833	0.787						
Music 5	0.859	0.813						
Art Mastery Motivation								
Art 6	0.869	0.932	0.935	0.935	0.706	0.706	0.935	0.937
Art 3	0.917	0.868						
Art 5	0.763	0.897						
Art 4	0.884	0.803						
Art 2	0.873	0.792						
Art 1	0.718	0.734						
English Mastery Motivation								
ENG3	0.893	0.888	0.921	0.893	0.662	0.573	0.922	0.889
ENG4	0.898	0.811						
ENG6	0.845	0.863						
ENG5	0.783	0.734						
ENG2	0.761	0.678						
ENG1	0.679	0.580						
Mathematics Mastery Motivation								
Math 4	0.799	0.825	0.902	0.903	0.605	0.605	0.901	0.901
Math 6	0.801	0.797						
Math 2	0.812	0.835						
Math 1	0.790	0.738						
Math 5	0.722	0.794						
Math 3	0.740	0.678						
Science Mastery Motivation								
Science 3	0.779	0.876	0.864	0.904	0.517	0.612	0.862	0.903
Science 2	0.776	0.807						
Science 1	0.768	0.788						
Science 5	0.715	0.737						
Science 4	0.641	0.716						
Science 6	0.618	0.758						
Reading Mastery Motivation								
Reading 4	0.720	0.670	0.876	0.854	0.542	0.497	0.878	0.865
Reading 2	0.813	0.760						
Reading 5	0.779	0.817						
Reading 3	0.776	0.722						
Reading 6	0.665	0.673						
Reading 1	0.650	0.560						

**Table 4:** HTMT ratio of correlations among the SSMMQ factors of the Romanian and Russian versions.

SSMMQ Scales	Music	Art	English	Mathematics	Science	Reading
Music		0.295	0.228	0.151	0.400	0.346
Art	0.398		0.164	0.183	0.439	0.335
English	0.172	0.154		0.458	0.312	0.424
Math	0.163	0.223	0.518		0.326	0.458
Science	0.418	0.406	0.378	0.449		0.536
Reading	0.441	0.486	0.437	0.518	0.597	

*Note*: The upper triangle contains the Russian data, the lower triangle presents the Romanian data.

**Table 5:** Fornell–Larcker criterion: Correlations between the square roots of the AVE of each variable.

SSMMQ Scales	Romanian						Russian					
	1	2	3	4	5	6	1	2	3	4	5	6
1. Music	**0.870**						**0.835**					
2. Art	0.404	**0.840**					0.299	**0.840**				
3. English	0.175	0.156	**0.814**				0.233	0.167	**0.757**			
4. Math	0.165	0.224	0.386	**0.778**			0.152	0.184	0.506	**0.778**		
5. Science	0.421	0.408	0.381	0.448	**0.719**		0.404	0.441	0.316	0.326	**0.782**	
6. Reading	0.448	0.491	0.443	0.521	0.600	**0.736**	0.356	0.343	0.438	0.467	0.547	**0.705**

*Note*: Average shared squared variance (in bold).

**Table 6:** Correlations of SSMMQ factors by language and grade.

SSMMQ Scales	Language		Grade Level		
	Romanian	Russian	5th Grade	7th Grade	9th Grade
Reading-Math	0.472 **	0.410 **	0.518 **	0.423 **	0.417 **
Reading-Science	0.520 **	0.468 **	0.532 **	0.383 **	0.488 **
Reading-English	0.438 **	0.417 **	0.391 **	0.474 **	0.415 **
Reading-Art	0.445 **	0.315 **	0.386 **	0.503 **	0.396 **
Reading-Music	0.410 **	0.297 **	0.410 **	0.432 **	0.321 **
Math-Science	0.391 **	0.293 **	0.454 **	0.306 **	0.275 **
Math-English	0.381 **	0.486 **	0.501 **	0.400 **	0.386 **
Math-Art	0.191 **	0.174 **	0.131 *	0.279 **	0.092
Math-Music	0.164 **	0.147 **	0.215 **	0.201 **	0.036
Science-English	0.368 **	0.291 **	0.315 **	0.205 **	0.347 **
Science-Art	0.367 **	0.408 **	0.365 **	0.331 **	0.342 **
Science-Music	0.394 **	0.379 **	0.314 **	0.446 **	0.397 **
English-Art	0.167 **	0.157 **	0.103	0.201 **	0.151 **
English-Music	0.187 **	0.243 **	0.209 **	0.205 **	0.205 **
Art-Music	0.394 **	0.285 **	0.347 **	0.413 **	0.331 **

*Note*: * *p* < 0.05; ** *p* < 0.01.

**Table 7:** Goodness of fit statistics: Baseline models.

Groups	Model	χ^2^ (df)	TLI	CFI	RMSEA [90% CI]	SRMR
Romanian	Original model	1433.384 (579)	0.897	0.889	0.062 [0.047, 0.054]	0.040
	Modified model	1090.799 (574)	0.963	0.966	0.039 [0.036, 0.043]	0.041
	Baseline model	1136.058 (574)	0.960	0.963	0.041 [0.037, 0.044]	0.042
Russian	Original model	1228.756 (579)	0.879	0.869	0.065 [0.052, 0.061]	0.050
	Modified model	985.752 (574)	0.948	0.953	0.045 [0.040, 0.050]	0.049
	Baseline model	1001.223 (574)	0.946	0.951	0.046 [0.041, 0.051]	0.048
5th grade	Original model	1028.426 (579)	0.896	0.888	0.058 [0.043, 0.052]	0.045
	Modified model	878.451 (575)	0.957	0.961	0.039 [0.034, 0.044]	0.045
	Baseline model	892.844 (574)	0.955	0.959	0.040 [0.035, 0.045]	0.046
7th grade	Original model	1102.555 (579)	0.893	0.884	0.064 [0.050, 0.060]	0.050
	Modified model	938.494 (576)	0.950	0.955	0.046 [0.040, 0.051]	0.051
	Baseline model	920.029 (574)	0.953	0.957	0.045 [0.039, 0.050]	0.050
9th grade	Original model	1294.335 (579)	0.855	0.844	0.075 [0.061, 0.070]	0.052
	Modified model	1006.727 (573)	0.940	0.945	0.051 [0.046, 0.056]	0.051
	Baseline model	1058.615 (574)	0.933	0.939	0.054 [0.049, 0.059]	0.051
Female	Original model	1283.040 (579)	0.888	0.880	0.063 [0.047, 0.054]	0.041
	Modified model	1024.173 (574)	0.958	0.961	0.041 [0.037, 0.045]	0.041
	Baseline model	1043.189 (574)	0.956	0.960	0.042 [0.038, 0.046]	0.041
Male	Original model	1322.864 (579)	0.884	0.874	0.063 [0.049, 0.056]	0.045
	Modified model	1054.240 (575)	0.953	0.957	0.042 [0.038, 0.046]	0.044
	Baseline model	1053.149 (574)	0.953	0.957	0.042 [0.038, 0.046]	0.044

**Table 8:** Measurement invariance models by language, grade, and gender.

Models	χ^2^	CFI	RMSEA [90% CI]	SRMR	ΔCFI	ΔRMSEA	ΔSRMR	Decision
Language invariance models (*N*_RO_ = 586, *N*_RU_ = 353)								
Configural	2564.612 (1146)	0964	0.029 [0.027, 0.030]	0.042				
Metric	2181.757 (1176)	0.958	0.030 [0.028, 0.032]	0.042	−0.006	0.001	0.000	Accept
Scalar	2081.155 (1212)	0.947	0.032 [0.030, 0.034]	0.046	−0.011	0.002	0.004	Reject
Scalar (Music 4)	2050.433 (1211)	0.949	0.032 [0.029, 0.034]	0.046	−0.009	0.002	0.004	Accept
Residual	2715.169 (1247)	0.939	0.035 [0.034, 0.037]	0.043	−0.010	0.003	−0.003	Accept
Grade level invariance models (*N*_5_ = 346, *N*_7_ = 304, *N*_9_ = 289)								
Configural	2887.185 (1719)	0.951	0.028 [0.026, 0.029]	0.051				
Metric	2906.709 (1779)	0.952	0.026 [0.024, 0.028]	0.047	0.001	−0.002	−0.004	Accept
Scalar	3185.840 (1851)	0.944	0.028 [0.026, 0.029]	0.048	0.008	0.002	0.001	Accept
Residual	3315.650(1923)	0.941	0.028 [0.026, 0.029]	0.048	0.003	0.000	0.000	Accept
Gender invariance models (*N*_FA_ = 472, *N*_MA_ = 466)								
Configural	2085.271 (1146)	0.959	0.030 [0.028, 0.032]	0.041				
Metric	2141.529 (1176)	0.958	0.030 [0.028 0.032]	0.042	−0.001	0.000	0.001	Accept
Scalar	2368.548 (1212)	0.949	0.032 [0.030, 0.034]	0.044	−0.009	0.002	0.002	Accept
Residual	2482.448 (1248)	0.946	0.032 [0.031, 0.034]	0.045	−0.003	0.000	0.001	Accept

**Table 9:** Latent mean differences for language, gender, and grade.

Groups	SSMMQ Scale	MD	CR	*d*
Gender ^1^	Music	−0.609	−7.064 ***	0.488
	Art	−0.902	−10.531 ***	0.683
	English	−0.284	−4.544 ***	0.334
	Math	−0.082	−1.392	
	Science	−0.287	−4.360 ***	0.295
	Reading	−0.415	−7.756 ***	0.531
Languages ^2^	Music	−0.302	−2.992 *	0.175
	Art	−0.436	−4.273 ***	0.013
	English	−0.122	−1.802	
	Math	−0.261	−3.761 ***	0.196
	Science	−0.174	−2.124 *	0.129
	Reading	−0.086	−1.353	
5th grade vs. 7th grade ^3^	Music	−0.297	−2.819 *	0.229
	Art	−0.474	−4.668 ***	0.379
	English	0.049	0.681	
	Math	−0.295	−4.216 ***	0.329
	Science	−0.107	−1.348	
	Reading	−0.015	−0.231	
5th grade vs. 9th grade	Music	−0.398	−3.729 ***	0.285
	Art	−0.809	−7.542 ***	0.427
	English	−0.220	−2.794 *	0.205
	Math	−0.178	−2.484 *	0.190
	Science	−0.220	−2.714 *	0.231
	Reading	−0.062	−0.943	
7th grade vs. 9th grade	Music	−0.101	−0.914	
	Art	−0.335	−2.935 ***	0.213
	English	−0.269	−3.313 ***	0.258
	Math	0.116	1.527	
	Science	−0.112	−1.355	
	Reading	−0.047	−0.687	

*Note*: ^1^ χ^2^ (*df*) = 2219.189 (1206), CFI = 0.956, TLI = 0.954, RMSEA = 0.030 [0.028, 0.032], SRMR = 0.042. ^2^ χ^2^ (*df*) = 2016.882 (1205), CFI = 0.951, TLI = 0.949, RMSEA = 0.031 [0.029, 0.033], SRMR = 0.047. ^3^ χ^2^ (*df*) = 3083.106 (1841), CFI = 0.948, TLI = 0.946, RMSEA = 0.027 [0.025, 0.028], SRMR = 0.047, * *p* < 0.05, *** *p* < 0.001.

## Data Availability

Data available on request due to privacy and ethical restrictions.
